# Multifocal Osteonecrosis in a Patient With Systemic Lupus Erythematosus and Antiphospholipid Syndrome Associated With Pyoderma Gangrenosum and Other Complications: A Case Report

**DOI:** 10.1155/carm/7107020

**Published:** 2026-02-23

**Authors:** Ehsan Adib, Shokofeh Banaei, Afsaneh Enteshari-Moghaddam

**Affiliations:** ^1^ Students Research Committee, School of Medicine, Ardabil University of Medical Sciences, Ardabil, Iran, arums.ac.ir; ^2^ Department of Physiology, School of Medicine, Ardabil University of Medical Sciences, Ardabil, Iran, arums.ac.ir; ^3^ Department of Internal Medicine, School of Medicine, Ardabil University of Medical Sciences, Ardabil, Iran, arums.ac.ir

**Keywords:** antiphospholipid syndrome, autoimmunity, multifocal osteonecrosis, pyoderma gangrenosum, systemic lupus erythematosus

## Abstract

Systemic lupus erythematosus (SLE) is a quintessential illustration of an autoimmune disease. It results from complex interactions that include impaired apoptotic clearance, increased activity of both the innate and adaptive immune systems, complement activation, the development of immune complexes, and ensuing tissue inflammation, which collectively contribute to a sustained autoimmune response. We present a case relating to a 37‐year‐old female patient (from Ardabil, Iran) who received a diagnosis of SLE and antiphospholipid syndrome (APS), in accordance with established diagnostic criteria. She is currently admitted to the hospital due to a recurring pyoderma gangrenosum ulcer, accompanied by pain and swelling in her left leg. The patient tested positive for ANA, lupus anticoagulant, anti–beta2‐glycoprotein I (anti–beta2GPI), and anti‐Ro antibodies. These results, along with her clinical manifestations such as spontaneous abortion, stroke, malar rash, photosensitivity, and pyoderma gangrenosum ulcers, confirm a diagnosis of SLE associated with APS according to the relevant criteria. After appropriate medical treatment, she was identified as experiencing a lupus flare and multifocal osteonecrosis in the knee, hip, and shoulder.

## 1. Introduction

Systemic lupus erythematosus (SLE) represents a classic example of an autoimmune disorder. It arises from a multifaceted interplay involving defective apoptotic clearance, heightened activity of both the innate and adaptive immune systems, complement activation, the formation of immune complexes, and subsequent tissue inflammation, leading to a persistent autoimmune response [1]. SLE susceptibility has a genetic basis, but it is widely recognized that environmental and other factors play crucial roles in triggering the onset of the disease. The severity of SLE varies among patients, ranging from mild skin manifestations to severe damage to vital organs, with outcomes that can range from prolonged remission to fatality. The diagnosis of SLE relies on distinctive clinical signs involving the skin, joints, kidneys, and CNS, along with serological indicators such as antinuclear antibodies (ANA), particularly those targeting double‐stranded DNA (dsDNA) [2]. The 2019 EULAR/ACR classification criteria established by the European League Against Rheumatism and the American College of Rheumatology are utilized for the diagnosis of SLE [3].

Antiphospholipid syndrome (APS) is characterized by the occurrence of venous, arterial, and small‐vessel thrombosis, which can result in osteonecrosis (ON), as well as complications such as pregnancy loss and preterm delivery, particularly in patients experiencing severe preeclampsia or placental insufficiency who have persistent antiphospholipid antibodies (aPL). Additional clinical features may include cardiac valvular disease, renal thrombotic microangiopathy, thrombocytopenia, hemolytic anemia, and cognitive deficits in individuals with aPL. The aPL consists of three primary antibodies: anticardiolipin (aCL), lupus anticoagulant (LA), and anti–beta2‐glycoprotein I antibodies (anti–b2GPI) [4, 5]. Furthermore, APS is frequently linked with other systemic autoimmune disorders, notably SLE [6].

## 2. Case Presentation

**FIGURE 1:**
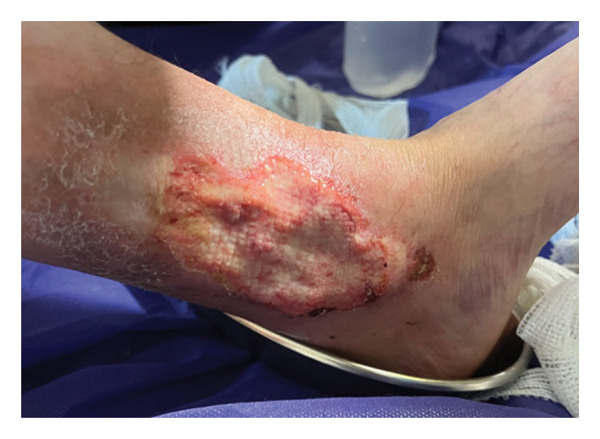
Pyoderma gangrenosum ulcer on the patient’s left leg on the day of hospitalization.

A 37‐year‐old female patient with a history of SLE and APS, who was diagnosed three years ago, was admitted to the hospital due to a recurrence of an infectious ulcer on her left leg that had begun two days earlier. This condition arose following irregular medication use and subsequent discontinuation, accompanied by pain throughout her left leg. The patient’s wound was a pyoderma gangrenosum (PG), for which an ulcer biopsy had previously been performed 3 years ago, revealing mixed cellular inflammation with neutrophil predominance, which ruled out the suspicion of venous ulceration. The ulcer exhibited a distinct red border with yellow‐white purulent discharge and was located on the upper portion of the inner malleolus of the left foot, measuring 5 × 9 cm (Figure 1).

**FIGURE 2:**
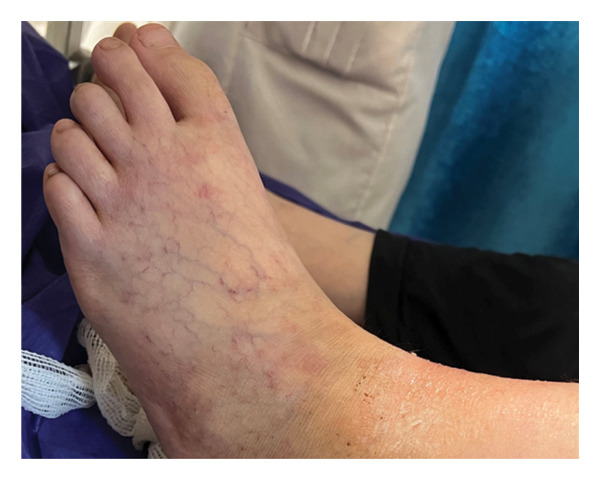
Livedo reticularis in the patient’s left foot.

She had a spontaneous abortion 8 years prior, which led to her inability to conceive thereafter. Furthermore, she has experienced multiple episodes of deep vein thrombosis (DVT) in both lower limbs over the last 4 years and has suffered a stroke 2 years prior, which fortunately did not lead to any complications. Two years ago, she presented with cardiac symptoms, including hypotension and shortness of breath, prompting an echocardiographic evaluation that resulted in a diagnosis of Libman–Sacks endocarditis. Her ejection fraction (EF) was 25%, indicating the presence of heart failure (HF). She tested positive for ANA, LA, anti–beta2GPI, and anti‐Ro antibodies. These results, along with her clinical manifestations, confirmed a diagnosis of SLE associated with APS according to the criteria. She has been treated with CellCept (mycophenolate mofetil), 15 mg of prednisolone, folic acid, and 80 mg of aspirin (ASA) for the past 3 years. Additionally, she was prescribed carvedilol and Aldactone (spironolactone) for her HF. The patient previously took warfarin 5 mg, but her adherence to this medication was inconsistent, and she ceased taking it 1 month ago. Vital signs recorded included a blood pressure of 110/75 mmHg, a heart rate of 77 beats per minute, a respiratory rate of 14 breaths per minute, a body temperature of 38°C, and an arterial oxygen saturation of 96%. Upon skin examination, livedo reticularis was observed in both lower limbs (Figure 2).

The pulse was symmetrical and normal in both upper limbs, although a weaker pulse was observed in the left lower limb than in the right lower limb. The examination revealed a restricted range of motion in knee flexion for both lower limbs, which was painful for the patient. The left knee was also swollen and tender to the touch. On the basis of this clinical presentation, the patient was admitted to the hospital, where daily wound dressing was implemented. Additionally, due to the patient’s history of multibacterial infections in this area of the wound, as well as the high suspicion of wound contamination by multiple bacteria, and until the results of the culture of the wound secretions were ready, the broad‐spectrum antibiotics vancomycin and imipenem were included in the patient’s medication plan. In light of knee joint swelling, increased fatigue, and the reappearance of PG ulcers, there was a high suspicion that the patient was experiencing an SLE flare due to inconsistent medication adherence. Consequently, a dexamethasone pulse was initiated for the patient. Hematological, serological, and immunological assessments were ordered, with the results detailed in Tables 1 and 2.

**TABLE 1:** Initial blood test results of the patient.

	Result	Normal range
WBC (10 ∗ 3/μL)	4.2	4–10
RBC (10 ∗ 6/μL)	3.56	4.2–5.4
HGB (g/dL)	7.4	12–16
HCT (%)	27.7	36–46
MCV (FL)	78	81–96
MCH (pg)	21	26–32
MCHC	27	26–36
Platelets (10 ∗ 3/μL)	422	150–450
ESR 1^st^h (mm/h)	80	0–15
PT (s)	14.9	—
INR	1.07	—
PTT (s)	36	30–45
Creatinine (mg/dL)	0.9	0.9–1.3
Ca (mg/dL)	10.3	8.5–10.5
K (mEq/L)	4	3.5–5.5

*Note:* HGB: hemoglobin, HCT: hematocrit, K: potassium, Ca: calcium.

Abbreviations: ESR, erythrocyte sedimentation rate; INR, international normalized ratio; MCH, mean corpuscular hemoglobin; MCHC, mean corpuscular hemoglobin concentration; MCV, mean corpuscular volume; PT, prothrombin time; PTT, partial thromboplastin time; RBC, red blood cell; WBC, white blood cell.

**TABLE 2:** Immunological results in the patient.

	Result	Normal range
C3	141	75–175
C4	20	15–45
P‐ANCA	4.3	0–18
C‐ANCA	3.9	0–18
Antiphospholipid antibody IgG	3.6	0–18
Anticardiolipin antibody IgG	2.7	0–18
Anticardiolipin antibody IgM	3.5	0–18
Antiphospholipid antibody	Negative	
Anti‐dsDNA	61	0–18
Anti–beta 2 glycoprotein IgM	4.1	> 12
Anti–beta 2 glycoprotein IgG	5.2	> 12

*Note:* dsDNA: double‐stranded DNA.

Abbreviation: ANCA, antineutrophil cytoplasmic antibodies.

**FIGURE 3:**
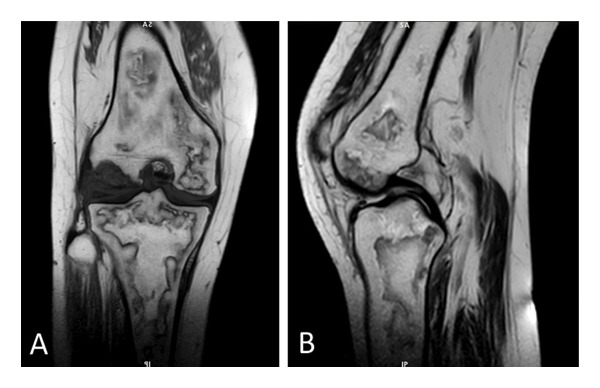
Left knee MRI showing osteonecrosis in the distal femur, proximal tibia, and fibula in the coronal (A) and sagittal views (B).

**FIGURE 4:**
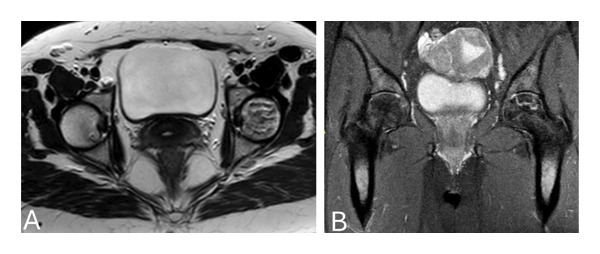
Hip MRI showing osteonecrosis in both femoral heads in axial (A) and coronal views (B).

**FIGURE 5:**
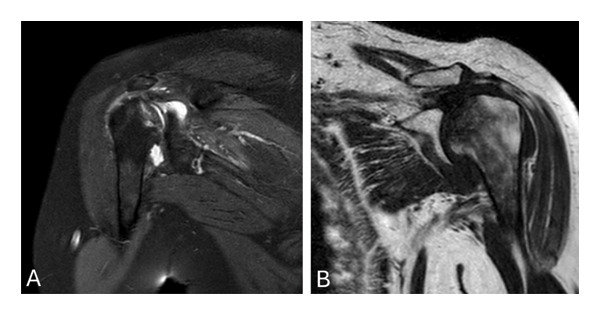
Shoulder MRI showing osteonecrosis of both the right (A) and left (B) heads of the humerus in the coronal view.

Owing to concerns regarding possible lower limb DVT, a bilateral Doppler ultrasound (US) of the lower limb vessels was performed, which revealed previous chronic DVT, with no acute thrombosis. Additionally, the arterial assessment revealed normal arterial flow. Furthermore, a moderate effusion was identified in the left knee. In light of the US findings and the patient’s complaints of knee pain, along with a high suspicion of ON, magnetic resonance imaging (MRI) of the left knee, hip, and shoulders was performed, which revealed ON in the distal femur, proximal tibia and fibula in the left knee (Figure 3), bilateral ON of femoral heads in the hip joints (Figure 4), and bilateral ON of the humerus head in the shoulders (Figure 5). The overall findings were indicative of multifocal osteonecrosis (MFON).

**FIGURE 6:**
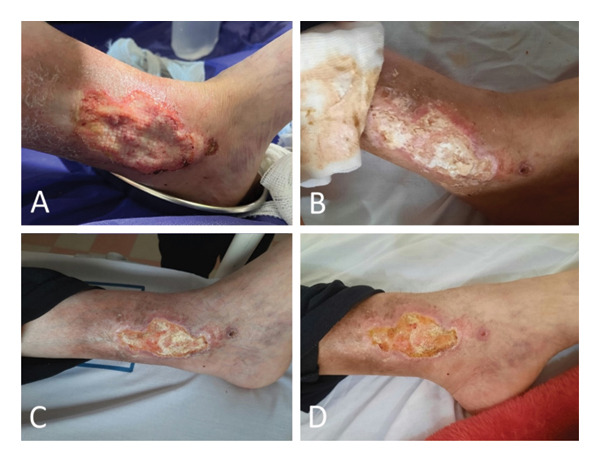
Pyoderma gangrenosum ulcer in the patient’s left leg. (A) First day of hospitalization. (B) Day 12. (C) Day 17. (D) Discharge day.

After the patient was hospitalized, she resumed taking medications that had been previously discontinued. To remove the patient from a lupus flare and prevent irreversible complications of this flare, despite knowing that corticosteroids may exacerbate ON, the prednisolone dosage was increased by 1 mg/kg until the flare subsided and the wound showed partial healing. At the earliest opportunity, the dosage was then gradually tapered to 15 mg to prevent MFON from worsening. The margin of the wound progressively became smaller, and the amount of purulent discharge widely decreased (Figure 6). She was discharged in good overall health from the hospital after 23 days, with the recommendation to consistently take her medications and not to discontinue them. She was in good overall health and was monitored at the rheumatology clinic every 3 months. She was also referred to the orthopedic clinic to evaluate her MFON and make decisions about possible related surgeries, such as joint replacement therapy (arthroplasty). Arthroplasty was not performed for this patient because of HF, a history of recurrent DVT, and a high risk of not being able to tolerate the surgery. Therefore, only conservative treatment was considered for the patient, with continued use of aspirin, anticoagulants, calcium, and vitamin D. After 3 months, the patient’s pain was reduced, and she took her medication regularly. Also, due to the patient’s symptoms being controlled and preventing the symptoms of MFON from worsening, the dose of prednisolone was tapered to 5 mg.

## 3. Discussion

In this case report, we describe a 37‐year‐old woman who was diagnosed with SLE and APS 3 years prior. She is presently hospitalized due to a recurrent PG ulcer, along with pain and swelling in her left leg and an SLE flare. Following appropriate medical interventions, she was diagnosed with MFON. SLE predominantly affects women of childbearing age, specifically those between 15 and 44 years of age, with a notable female‐to‐male ratio of 9:1, establishing SLE as one of the most sex‐specific autoimmune disorders [7]. The diverse clinical manifestations and underlying mechanisms of SLE contribute to the complexity of accurately defining this condition. Although new classification criteria have been developed for research, they cannot replace clinical judgment in diagnosing SLE [3]. Even with progress in the management of diseases, individuals suffering from SLE still face considerable risks of morbidity and mortality. Factors such as persistent disease activity, comorbidities, and drug toxicity greatly increase the likelihood of irreversible damage and increased mortality [8, 9]. Life expectancy may be adversely affected by significant organ involvement, particularly in the kidneys, lungs, and heart. However, with diligent monitoring, approximately 80%–90% of SLE patients can expect an average life span [10]. APS is particularly significant in the context of SLE, affecting between 6% and 30% of patients in various studies and contributing to notable morbidity and mortality rates [11]. It is crucial to assess the overall risk of thrombotic events in patients with APS by considering both clinical and laboratory data. Furthermore, the presence of SLE is viewed as a higher risk factor for these patients. Therefore, when a patient with SLE experiences a thrombotic event, it is clinically significant due to its potentially life‐threatening nature, its impact on quality of life, and the challenges it poses for clinicians [12].

PG is a chronic and recurring neutrophilic dermatosis characterized by the development of painful, rapidly expanding skin ulcers. PG usually starts as sterile pustules that swiftly develop into erythematous to violaceous ulcerative plaques, characterized by irregular and undermined margins, a violaceous border, and central purulent or hemorrhagic fluid [13]. The underlying mechanisms of PG are complex and involve a marked dysregulation of both innate and adaptive immune responses, with the follicular unit being increasingly recognized as the probable initial target [14]. PG predominantly affects individuals aged 20–50 years, with a higher prevalence in women. The ulcerative lesions associated with this condition can manifest on any part of the body, although they are most frequently found on the lower extremities [15]. There is a well‐documented correlation between PG and various immune‐mediated disorders, particularly inflammatory bowel disease, rheumatoid arthritis, and systemic sclerosis, which are among the most common comorbidities. However, the occurrence of SLE in conjunction with PG is quite rare [16]. The advancement of effective treatment options for PG has been limited by studies that provide limited evidence and the lack of validated criteria for diagnosis and treatment response [14]. The diagnosis of PG presents significant challenges owing to the absence of standardized clinical diagnostic criteria, which hinders prompt identification. Early identification of PG is crucial for initiating treatment and reducing the risk of developing large, necrotic, and highly painful ulcers that may become secondarily infected, resulting in disfiguring scars. Given that the pathophysiology of PG is linked to immune system abnormalities, immunosuppressive therapy is considered the primary treatment approach [17].

ON or avascular necrosis (AVN) is characterized by the necrosis of bone tissue caused by inadequate blood supply, potentially leading to joint pain, bone destruction, difficulty in mobility, and a decrease in functional performance [18]. Despite extensive research, there is no established consensus regarding its etiology or pathogenesis. However, the development of ON has been correlated with several risk factors, including the use of corticosteroids, alcohol misuse, smoking, and various inflammatory arthropathies, including SLE [19]. Corticosteroid therapy is the most significant factor linked to the onset of ON in patients with SLE [20]. In patients diagnosed with SLE, the prevalence of ON fluctuates between 3% and 44% [21]. It is hypothesized that SLE elevates the risk of ON due to a hypercoagulable state; one study indicated that SLE patients with ON had a 32% higher prevalence of various coagulation abnormalities than did the control group. Furthermore, these individuals are susceptible to APS, which may also contribute to the development of ON [22]. The femoral head (ONFH) is the most commonly affected site, followed by the knee, shoulder, and ankle, with instances of involvement in the elbow, wrist, and foot [23]. MFON, which affects more than three separate anatomic sites concurrently or consecutively, for example, ON of the hip, knee, and shoulder, or of the shoulder, knee, and ankle, is observed in approximately 3% of SLE patients. The diagnosis of ON in SLE patients is primarily established through radiographic findings following clinical suspicion [19, 24]. MRI is recognized as the gold standard for diagnosing both symptomatic and asymptomatic ON [25]. The treatment of MFON remains controversial; however, early‐stage management typically includes conservative treatments such as anticoagulants, statins, bisphosphonates, and core decompression, whereas arthroplasty is reserved for advanced cases [24, 26]. Case reports of MFON are limited, and many MFON patients are asymptomatic, making the diagnosis difficult. Therefore, the exact incidence of MFON among patients with different diseases is unknown [24]. Physicians should note that SLE patients’ limb pain (like in our patient) is not always due to SLE arthritis. There is always a risk that these patients will develop ON or even MFON in the lower and upper limbs due to hypercoagulable conditions of SLE or even high‐dose corticosteroid therapy, and physicians should be able to confirm their diagnosis by performing an MRI if clinically suspected.

## Author Contributions

E.A. and A.E‐M. conceived the idea for the paper, drafted the original manuscript, and supervised the project. S.B. reviewed and edited the final manuscript. And finally, all the authors contributed to the manuscript.

## Funding

The authors received no financial support for the research, authorship, and/or publication of this article.

## Disclosure

All the authors read and approved the final manuscript.

## Ethics Statement

The work was approved by the Ethics Committee of Biomedical Research, Ardabil University of Medical Sciences, which issued the approval number IR.ARUMS.REC.1403.438.

## Consent

Written informed consent was obtained from the patient for publication of this case report.

## Conflicts of Interest

The authors declare no conflicts of interest.

## Data Availability

The data that support the findings of this study are available from the corresponding author upon reasonable request.
